# A simple suspension culture method for generating human iPSC-derived liver organoids

**DOI:** 10.1093/biomethods/bpag036

**Published:** 2026-06-25

**Authors:** Ryosuke Morozumi, Mawo Kinoshita, Ryo Takahashi, Masayuki Mishima, Kazuki Izawa, Kei-ichi Sugiyama, Masataka Tsuda

**Affiliations:** Division of Genome Safety Science, National Institute of Health Sciences, 3-25-26 Tonomachi, Kawasaki-ku, Kawasaki, Kanagawa, 210-9501, Japan; Division of Genome Safety Science, National Institute of Health Sciences, 3-25-26 Tonomachi, Kawasaki-ku, Kawasaki, Kanagawa, 210-9501, Japan; Division of Genome Safety Science, National Institute of Health Sciences, 3-25-26 Tonomachi, Kawasaki-ku, Kawasaki, Kanagawa, 210-9501, Japan; Laboratory of Veterinary Toxicology, Cooperative Department of Veterinary Medicine, Tokyo University of Agriculture and Technology, 3-5-8, Saiwai-cho, Fuchu-shi, Tokyo, 183-8509, Japan; Tsukuba Research Institute, BoZo Research Center Inc., 8 Okubo, Tsukuba-shi, Ibaraki, 300-2611, Japan; Division of Genome Safety Science, National Institute of Health Sciences, 3-25-26 Tonomachi, Kawasaki-ku, Kawasaki, Kanagawa, 210-9501, Japan; Division of Genome Safety Science, National Institute of Health Sciences, 3-25-26 Tonomachi, Kawasaki-ku, Kawasaki, Kanagawa, 210-9501, Japan; Division of Genome Safety Science, National Institute of Health Sciences, 3-25-26 Tonomachi, Kawasaki-ku, Kawasaki, Kanagawa, 210-9501, Japan; Division of Genome Safety Science, National Institute of Health Sciences, 3-25-26 Tonomachi, Kawasaki-ku, Kawasaki, Kanagawa, 210-9501, Japan

**Keywords:** human liver organoid, human iPS cells, suspension culture, cytochrome P450 3A4

## Abstract

Human liver *in vitro* models are indispensable for toxicity studies requiring metabolic competence. However, widely used two-dimensional hepatocyte-derived cell lines exhibit limited drug-metabolizing capacity, whereas primary human hepatocytes are constrained by limited availability, donor variability, dedifferentiation, and poor long-term functional stability. Although three-dimensional liver models offer greater physiological relevance, many existing methods depend on exogenous extracellular matrix (ECM) scaffolds or specialized equipment, thereby limiting standardization, scalability, and broader implementation. Here, we developed a simple, scalable suspension culture method for generating human induced pluripotent stem cell (iPSC)-derived liver organoids (HLOs) without exogenous ECM scaffolds or specialized equipment. The resulting HLOs secreted albumin and exhibited hepatic gene expression profiles by RNA-seq analysis. Notably, cytochrome P450 3A4 activity was maintained more stably over an extended culture period than under the ECM-embedding condition. Immunostaining further confirmed the presence of hepatocyte-like populations together with CK19-positive epithelial structures exhibiting E-cadherin-positive cell–cell adhesion and ZO-1-positive tight junction-associated regions. These findings indicate that the HLOs recapitulate key structural and functional features of human liver tissue. Collectively, our results demonstrate that this simple suspension culture method provides a practical and accessible strategy for generating HLOs with sustained hepatic functionality. This platform should facilitate broader application of HLOs in drug screening, toxicity assessment, disease modeling, and other *in vitro* settings requiring reproducible and scalable human liver models.

## Introduction

Human cell-based *in vitro* models have been widely used to recapitulate hepatic metabolism. However, hepatocyte-derived cell lines maintained under two-dimensional culture conditions, such as HepG2, generally exhibit low expression and activity of major drug-metabolizing enzymes, particularly cytochrome P450 enzymes including CYP3A4, thereby limiting their suitability for metabolism-dependent toxicity assessment [1]. Primary human hepatocytes (PHHs), although they possess relatively high metabolic capacity, remain subject to persistent limitations, including restricted availability, donor-to-donor variability, dedifferentiation during culture, and difficulty in maintaining long-term hepatic function [2–4]. In particular, alterations in gene expression networks and functional decline associated with two-dimensional culture may compromise the reproducibility and predictive performance of toxicity responses [2]. Collectively, these constraints hinder the establishment of stable toxicity assessment platforms with high human relevance [4]. To improve metabolic competence, human cell systems with more liver-like drug metabolism have been explored. HepaRG cells have attracted attention as a more physiologically relevant human liver model because they can exhibit higher metabolic capacity than conventional hepatocyte-derived cell lines [5–8]. Nevertheless, their functional performance and range of applicability remain limited, underscoring the need for new *in vitro* liver models that complement existing systems [9, 10]. As an alternative strategy, human CYP enzymes have been introduced into the human lymphoblastoid cell line TK6 to confer metabolic activation capacity [11].

In parallel, recent advances in three-dimensional culture technologies have brought liver spheroids, human liver organoids (HLOs), and microphysiological systems (MPSs) to the forefront as models capable of better maintaining hepatic metabolic functions [12–17]. Under three-dimensional culture conditions (SCs), cell–cell interactions and the local microenvironment can be reconstituted in a manner that more closely resembles the *in vivo* state, thereby favoring the maintenance of albumin secretion, cytochrome P450 activity, bile acid-related functions, and other hepatic properties. In particular, liver models derived from human pluripotent stem cells (PSCs), especially induced pluripotent stem (iPS) cells, hold considerable promise as future standardized platforms because of their stable supply and reproducibility [18–21]. Moreover, active efforts are being made to incorporate defined cellular compositions, maturation strategies, and disease-associated contexts, such as steatosis and inflammation, into these systems [22–25]. Studies aimed at reproducing liver-specific functions, including bile acid transport and cholestatic responses, are likewise progressing [26, 27]. At the same time, increasing attention has been directed toward enhancing physiological complexity through the incorporation of immune cells, stromal cells, and vascular components, as reflected by the accumulation of methods incorporating Kupffer cells and approaches that promote vascularization and maturation [28–30]. These human PSC-derived HLOs span a range of structural complexity and can be broadly classified into three configurations [31]: epithelial-type organoids [32], multi-tissue organoids [21, 24], and multi-organ organoids [33]. Epithelial-type organoids are relatively simple and advantageous for expansion and handling, whereas multi-organ systems provide higher-order complexity at the expense of interpretability and practical simplicity. By contrast, multi-tissue HLOs represent an intermediate format that combines hepatic relevance with structural features such as biliary-like organization. In this context, the previously reported multi-tissue configuration [21] provides a useful reference for considering how structural complexity and practical tractability may be balanced in HLO design.

Despite these advances, most currently available HLO culture methods remain dependent on exogenous extracellular matrix (ECM) scaffolds such as Matrigel. This dependence becomes particularly problematic when structurally complex organoid formats, such as multi-tissue HLOs, are considered for routine implementation in toxicity testing and related applications. Although exogenous ECM scaffolds facilitate three-dimensional tissue organization, they have several well-recognized drawbacks, including lot-to-lot variability, undefined composition, effects on molecular diffusion and exposure uniformity, difficulty in scale-up and standardization, and high cost, all of which may constrain their practical use in toxicity testing [19, 34, 35]. Recent findings further suggest that the functions provided by exogenous ECM scaffolds, including adhesion, mechanical support, and ligand presentation, should be dissected and replaced with defined materials, particularly because such scaffolds may differentially influence the generation of distinct classes of HLOs [36]. In response to these limitations, alternative approaches have been reported, including suspension culture with low concentrations of ECM in the medium [32], self-organizing iPSC-derived liver organoids generated without ECM, and scalable ECM-free culture systems [37, 38]. However, such ECM-free approaches may still depend on specialized equipment or specialized culture, agitation, or supply systems, such as bioreactors, microfluidic platforms, or customized plates and perfusion systems. Accordingly, when broad implementation or multi-site use is envisaged, ease of adoption, including equipment requirements, technical demands, and operational burden, becomes a critical practical consideration [14, 15, 39, 40]. These considerations highlight the importance of developing practical approaches for generating multi-tissue liver organoids.

In the present study, we established a simple suspension culture method for generating human iPSC-derived liver organoids based on a multi-tissue HLO concept. This method does not require exogenous ECM scaffolds or specialized equipment. Organoids generated using this method exhibited albumin secretion and liver-like molecular characteristics in RNA-seq analysis. Moreover, CYP3A4 activity was maintained over an extended culture period more stably than under the ECM-embedding condition. Immunostaining further confirmed the presence of hepatocyte-like populations together with biliary-like structures positive for ZO-1 and E-cadherin, while Desmin-positive and CD68-positive cells were detected only in small numbers. Taken together, these findings demonstrate that our method enables the generation of organoids possessing key structural and functional features of human liver tissue. This method therefore provides a practical approach for generating human iPSC-derived liver organoids and may prove useful for a broad range of *in vitr*o applications, including toxicity assessment.

## Materials and methods

### Cell culture

The human induced PSC (iPSC) line Cellartis human iPS cell line 18 (ChiPSC18) was used throughout this study [41]. ChiPSC18 cells were obtained from Takara Bio Inc. (Shiga, Japan) and maintained using the Cellartis DEF-CS Culture System (Y30010, Takara Bio Inc.) according to the manufacturer’s instructions. For routine passaging every 3–5 days, cells were dissociated with 1× TrypLE Select (Thermo Fisher Scientific, Waltham, MA, USA) for 5–7 min at 37°C and seeded at a density of 2–5 × 10^4^ cells/cm^2^ onto culture dishes coated with DEF-CS COAT-1 in DEF-CS medium.

### Differentiation of human iPSCs into HLOs

Human iPSCs were differentiated into definitive endoderm based on a previously described protocol with minor modifications [21, 24]. Human iPSCs were dissociated with TrypLE Select and seeded at a density of 1 × 10^5^ cells/cm^2^ onto 24-well tissue culture plates (Falcon #353047, Corning Inc., NY, USA) or 6-well plates (Costar #3736, Corning Inc., NY, USA). When the cells reached approximately 90%–95% confluency, differentiation was initiated by replacing the medium as follows. On day 1, cells were cultured in RPMI 1640 supplemented with 100 ng/ml Activin A and 50 ng/ml BMP4. On day 2, the medium was changed to RPMI 1640 containing 100 ng/ml Activin A and 0.2% fetal calf serum (FCS), and on day 3 to RPMI 1640 containing 100 ng/ml Activin A and 2% Knockout SR. From days 4 to 6, cells were cultured in Advanced DMEM/F12 supplemented with 2% B-27, 1% N-2, 500 ng/ml FGF4, 2 mM L-glutamine, 10 mM HEPES, and 3 μM CHIR99021. Cultures were maintained at 37°C in 5% CO_2_ with daily medium replacement. Spheroids formed by day 6 of differentiation. For HLO induction, spheroids and attached cells were gently harvested by pipetting on day 6 and centrifuged at 300 rpm for 3 min. After supernatant removal, cells in the ECM-embedding condition (EE) were embedded in 50 μL of 100% Geltrex and plated onto new culture plates (VWR #10062-896), followed by culture for 4 days in Advanced DMEM/F12 supplemented with B27, N2, and 2 μM retinoic acid (RA). In the suspension SC, cells were resuspended in fresh medium and transferred to new culture plates (VWR #10062-892 or #10062-896). After RA treatment, cultures were maintained in PowerPrimary HEP medium (PPM) supplemented with HGF (10 ng/ml), Dex (0.1 μM), and OSM (20 ng/ml). HLO induction cultures were maintained at 37°C in a humidified atmosphere containing 5% CO_2_, and the medium was changed every 3 days. Detailed information on the media and supplements used, including manufacturers and catalog numbers, is provided in Table S1.

### Whole-mount immunostaining of organoids

Whole-mount immunostaining of organoids was performed with minor modifications based on a previously published protocol [42]. HLO samples were collected on day 30. Organoids were harvested from suspension cultures, rinsed with phosphate-buffered saline (PBS), and fixed in 4% paraformaldehyde on ice for 45 min. After fixation, the organoids were washed with PBS containing 0.1% Tween-20 and permeabilized in a buffer containing 0.2% bovine serum albumin (BSA), 0.1% Triton X-100, and 0.01% sodium dodecyl sulfate (SDS). The permeabilized organoids were then blocked in blocking buffer (PBST containing BSA) for 1–2 h at room temperature and incubated with primary antibodies diluted in organoid washing buffer at 4°C overnight. Following washing, the organoids were incubated again in the same blocking buffer for 2 h at room temperature and subsequently treated with the appropriate fluorophore-conjugated secondary antibodies for 2 h at room temperature in the dark. Nuclei were counterstained with DAPI solution (Dojindo, 340-07971) at a dilution of 1:2000. Detailed information for all primary and secondary antibodies, including the antibody name, manufacturer, catalog number, and dilution, is provided in Table S2. The stained organoids were mounted in antifade mounting medium (Fluoromount™, Diagnostic BioSystems, K024), and immunofluorescence images were acquired using an Olympus FLUOVIEW FV3000 laser scanning confocal microscope system mounted on an Olympus IX83 inverted microscope. Image acquisition was performed using FV31S-SW software version 2.5.1. Images were processed using CellSens Dimension Desktop. Because images were acquired directly using the laser scanning confocal microscope system, no separate external camera was used for image acquisition.

### Functional assessment of organoids

Albumin secretion was quantified using a Human Albumin ELISA Kit (Bethyl Laboratories, E88-129) according to the manufacturer’s instructions. Culture supernatants were collected at the indicated time points or albumin measurement, and albumin levels were converted to secretion rates (ng/ml/day) based on the time interval between collections. Cytochrome P450 activity was assessed using the P450-Glo CYP3A4 Assay and Screening System (Promega, V9002) according to the manufacturer’s instructions. Culture supernatants were collected and subjected to the assay. In parallel, cellular ATP levels were measured from an aliquot of the same organoid suspension using the CellTiter-Glo 3D Cell Viability Assay (Promega, G9681) according to the manufacturer’s instructions. Absorbance and luminescence were measured using a TriStar^2^ LB 942 Multimode Microplate Reader (Berthold Technologies, Bad Wildbad, Germany). CYP3A4 activity was expressed as CYP3A4-dependent luminescence normalized to ATP-derived luminescence as an indicator of viable cell content, and values were scaled by 10^6^ for presentation. To compare temporal stability between SCs, the coefficient of variation of the mean CYP3A4 activity across days 30, 40, and 50 was calculated for each condition. The raw data used for albumin secretion and CYP3A4 activity analyses are provided in Tables S3 and S4, respectively.

### Statistics

Statistical analyses were performed using R version 4.5.2. Albumin secretion and CYP3A4 activity were analyzed by two-way analysis of variance ANOVA with SC and day as factors. When a significant SC × day interaction was detected, post hoc comparisons between SC and EE at each time point were performed using estimated marginal means with Bonferroni adjustment for multiple comparisons using the emmeans package. Out-of-range values were treated as missing values and excluded from statistical analyses. *P-*value < .05 were considered statistically significant.

### RNA sequencing and data analysis

Bulk RNA sequencing was performed using three biological replicates for each group (iPS and HLO). HLO samples were collected on day 25. Total cellular RNA was isolated using the NucleoSpin RNA kit (Takara Bio, #740955) according to the manufacturer’s protocol, and RNA concentration was measured using a Qubit fluorometric assay (Thermo Fisher Scientific Inc.). Library preparation and sequencing were outsourced to Azenta Life Sciences (Azenta Inc., USA). RNA-seq libraries were prepared according to the provider’s standard protocols and sequenced on the DNBSEQ platform to generate paired-end reads (2 × 150 bp). Raw reads were processed by adapter trimming and removal of low-quality bases (Phred score < 20), and reads shorter than 75 bp after trimming were discarded. Clean reads were aligned to the human reference genome (Ensembl GRCh38 release 101; Homo_sapiens.GRCh38.dna.toplevel) using HISAT2 (v2.2.0) with the corresponding gene annotation file (Homo_sapiens.GRCh38.101.gtf). Approximately 21–27 million reads were obtained per sample, with ∼95% of bases above Q30, and mapping rates exceeded 97% in all samples. Gene-level expression values and differential expression output files were obtained from the Azenta analysis pipeline. The raw sequencing data and processed RNA-seq data generated in this study have been deposited in the Gene Expression Omnibus under accession number GSE333276, and the processed data and downstream analysis scripts are also available in Zenodo: 10.5281/zenodo.19480024 [43].

### Heatmap analysis

Heatmap analysis was performed using a curated set of 49 genes of interest (GOI) selected to evaluate hepatic differentiation status and functional characteristics. The GOI set included markers of PSCs, transcription factors involved in hepatocyte differentiation and maturation, liver-specific functional genes, drug-metabolizing enzymes, bile acid and drug transporters, cholangiocyte markers, and genes associated with liver-resident cell populations, including hepatic stellate cells and Kupffer cells. Gene selection was based on previously published literature and established hepatic marker genes. Gene expression values were transformed using log2(TPM + 1) and *Z*-score normalized for each gene to enable comparison across samples. Heatmaps were generated using the pheatmap package in R(v4.5.2). Gene order was fixed according to predefined cell-lineage and functional categories without hierarchical clustering of rows, whereas hierarchical clustering was applied to columns.

### Gene ontology enrichment analysis

Gene ontology (GO) enrichment analysis was re-performed in R (v4.5.2) using the clusterProfiler package (v4.18.4), together with org. Hs.eg.db (v3.22.0), GO.db (v3.22.0), and enrichplot (v1.30.4). HLO-upregulated genes, defined as genes with log2 fold change ≥ 1.0 and recalculated Benjamini–Hochberg FDR < 0.05, were used as the input gene set. All genes included in the differential expression file were used as the background universe. Gene identifiers were converted from gene symbols to Entrez IDs prior to analysis. Enrichment was assessed separately for the biological process (BP) and cellular component (CC) ontologies using the enrichGO function with Benjamini–Hochberg multiple testing correction. Terms with adjusted *P-*value < .05 were considered significantly enriched. Enrichment results were visualized as dot plots showing the top-ranked terms by adjusted *P-*value.

### Differential gene expression analysis and visualization

Differential expression between iPS and HLO samples was evaluated using the differential expression output file generated by the Azenta pipeline. For downstream analysis, false discovery rates (FDRs) were recalculated from the reported *P-*value using the Benjamini–Hochberg method in R(v4.5.2). Genes with log2 fold change ≥ 1.0 and recalculated FDR < 0.05 were defined as HLO-upregulated genes. Volcano and MA plots were generated to visualize differential expression patterns between iPS and HLO samples. Volcano plots display log2 fold change versus −log10(FDR), whereas MA plots depict average expression levels and log2 fold change between HLO and iPS samples. Annotated genes were selected from the GOI set used in the heatmap analysis.

## Results

### Generation of human iPSC-derived liver organoids by a simple suspension culture method

**Figure 1:**
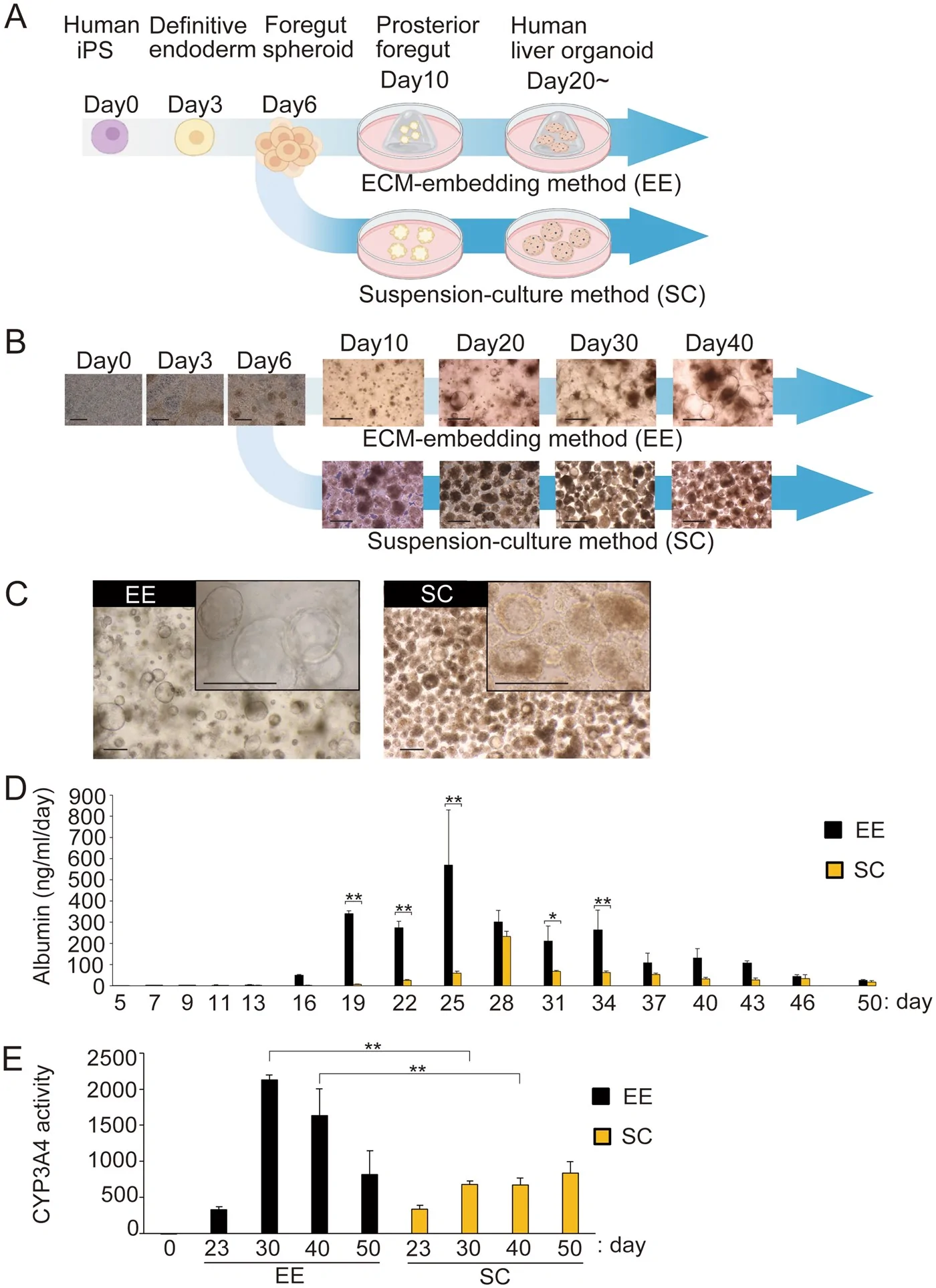
Generation and morphological characterization of HLOs. (**A**) Schematic overview of HLO differentiation. Human iPSCs were differentiated into definitive endoderm (day 3), foregut spheroids (day 6), posterior foregut (day 10), and subsequently into human liver organoids (HLOs; day 20 onward). Two culture conditions were applied: ECM-embedding (EE) and suspension culture (SC). (**B**) Bright-field images during differentiation. Representative images showing morphological changes during HLO differentiation under EE and SC conditions at the indicated time points. Scale bar, 500 µm in all panels. (**C**) Morphology of HLOs at later stages. Representative bright-field images of HLOs on day 23 cultured under EE and SC conditions. Insets show higher magnification views. Scale bar, 500 µm in all panels. (**D**) Albumin secretion during differentiation. Albumin secretion, expressed as ng/ml/day based on the culture supernatant collection interval, was measured over time under EE and SC conditions. Data are presented as mean ± SD of independent experiments. (**E**) CYP3A4 activity during differentiation. CYP3A4 activity was measured at the indicated time points and expressed as CYP3A4-dependent luminescence normalized to ATP-derived luminescence, an indicator of viable cell content, under EE and SC conditions. Data are presented as mean ± SD. The temporal coefficient of variation across days 30–50 was 43.3% for EE and 12.6% for SC. Statistical significance for panels D and E was assessed by two-way ANOVA with culture condition and day as factors, followed by Bonferroni-adjusted post hoc comparisons between SC and EE at each time point using estimated marginal means. Significance marks indicate corrected day-wise comparisons between EE and SC. *P < .05, **P < .01.

We first examined whether human iPSC-derived HLOs could be generated using a previously reported ECM-embedding method [21, 24], hereafter abbreviated as EE (upper panel of Fig. 1A). Briefly, human iPSCs were differentiated into definitive endoderm and subsequently into foregut spheroids, which were collected on day 6 and then subjected to the EE method. As previously reported, this approach yielded organoids measuring approximately 80–450 μm in diameter (upper panel of Fig. 1B). Higher-magnification imaging further confirmed that these organoids reproducibly exhibited the typical three-dimensional architecture (left panel of Fig. 1C). We next established a simple suspension culture method that does not require exogenous ECM scaffolds or specialized equipment. In this approach, human iPSCs were differentiated to the foregut spheroid stage using the same procedure as in the EE method, but were thereafter maintained in suspension in VWR culture dishes without ECM embedding or mechanical agitation such as shaking; this method is hereafter abbreviated as SC (lower panel of Fig. 1A). Under these conditions, organoid formation was also clearly observed (lower panel of Fig. 1B). Representative bright-field images at additional time points are shown in Fig. S1 (see online supplementary material). Notably, compared with the EE method, the SC method generated organoids appeared to exhibit a more compact internal cellular architecture (right panel of Fig. 1C). To assess hepatocyte-like function, albumin secretion into the culture supernatant was measured over time. Albumin secretion was detected under both conditions. Although secretion levels were generally higher under the EE condition, particularly between days 19 and 34, albumin secretion under the SC condition was maintained even after day 30 (Fig. 1D). These findings indicate that the SC method supports the formation of HLOs with hepatocyte-like secretory activity. We next assessed hepatic metabolic function by measuring CYP3A4 activity using a luminescence-based assay. Under the EE condition, CYP3A4 activity reached a high level at day 30 but subsequently declined at days 40 and 50. In contrast, under the SC condition, CYP3A4 activity was low at day 23 but was maintained thereafter through days 30, 40, and 50, indicating relatively stable activity during long-term culture (Fig. 1E). Consistent with this pattern, the temporal coefficient of variation of CYP3A4 activity across days 30–50 was lower under the SC condition than under the EE condition (12.6% vs. 43.3%), supporting more stable maintenance of CYP3A4 activity under SC. These results indicate that the SC and EE conditions showed distinct temporal profiles of hepatocyte-like functional readouts. Albumin secretion was detected under both conditions, although the EE condition generally showed higher secretion levels than the SC condition at several time points. For CYP3A4 activity, the EE condition produced a higher mean peak around day 30 followed by a subsequent decline, whereas the SC condition showed lower peak activity but relatively stable maintenance during extended culture. Two-way ANOVA revealed significant effects of SC, day, and the SC × day interaction for both albumin secretion and CYP3A4 activity. Bonferroni-adjusted post hoc comparisons using estimated marginal means showed significant differences between EE and SC at several time points for albumin secretion and at days 30 and 40 for CYP3A4 activity.

### Transcriptomic analysis reveals acquisition of hepatic molecular features in HLOs generated by the SC method

**Figure 2:**
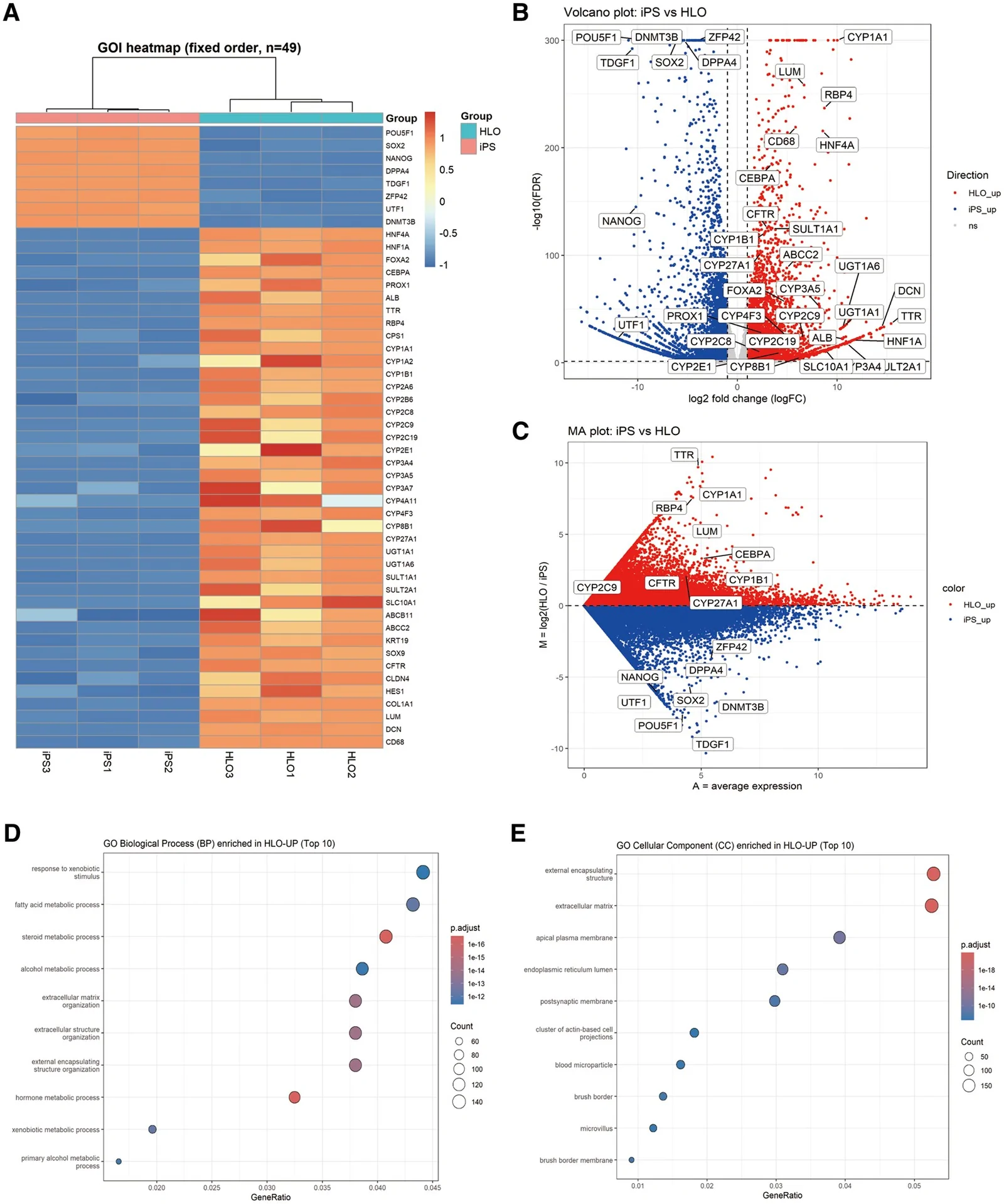
Transcriptomic characterization of hiPSCs and HLOs. (**A**) Heatmap of genes of interest (GOI). Heatmap showing expression patterns of 49 curated genes associated with pluripotency, hepatic differentiation and maturation, liver-specific functions, drug metabolism (phase I and II), transporters, and liver-resident cell populations. Gene expression values were log2(TPM + 1)-transformed and *Z*-score normalized. Genes were arranged in a predefined order without hierarchical clustering of rows, whereas hierarchical clustering was applied to samples. (**B**) Volcano plot of differential gene expression. Volcano plot showing differential gene expression between human iPSCs and HLOs. The *x*-axis represents log2 fold change (log2FC; HLO/human PSC), and the *y*-axis represents −log10 false discovery rate (FDR). Genes significantly upregulated in HLOsand those upregulated in human PSCs are indicated separately (|log2FC| ≥ 1, FDR < 0.05). Selected genes from the GOI set were annotated using more stringent criteria. (**C**) MA plot of gene expression. MA plot showing the relationship between average expression (A; mean TPM) and log2 fold change (M; log2[HLO/hiPSC]). Differentially expressed genes are highlighted as in (B), and selected GOI genes are annotated. (**D**) Gene ontology (GO) enrichment analysis (biological process). GO enrichment analysis of genes significantly upregulated in HLOs (FDR < 0.05), showing enriched biological process terms. (**E**) GO enrichment analysis (cellular component). GO enrichment analysis of genes significantly upregulated in HLOs (FDR < 0.05), showing enriched cellular component terms. Gene ratio and FDR-adjusted *P*-values are indicated by dot size and color, respectively.

To define the hepatic molecular features of HLOs generated by the SC method, we compared their transcriptomic profiles with those of human iPSCs by RNA-seq using three independent samples for each group. A heatmap generated from 49 GOI clearly separated HLOs from human iPSCs and revealed distinct expression patterns in the two groups (Fig. 2A). Human iPSCs showed high expression of pluripotency-associated genes, including POU5F1, SOX2, NANOG, and DPPA4, whereas HLOs exhibited increased expression of hepatic transcription factors (HNF4A, HNF1A, FOXA2, and CEBPA), liver functional genes (ALB, TTR, RBP4, and CPS1), and drug metabolism-related genes (CYP2C9, CYP2C19, CYP3A4, CYP3A5, UGT1A1, and ABCC2). Biliary epithelial-associated genes, such as KRT19, SOX9, and CFTR, were also upregulated, whereas several non-parenchymal cell-associated markers, including CD68, LUM, and DCN, were present at relatively lower levels. These findings indicate that HLOs generated by the SC method predominantly acquire hepatocyte-like molecular features together with a partial biliary epithelial signature. Consistent with these observations, the volcano plot showed upregulation of hepatic genes in HLOs, whereas genes associated with the undifferentiated state remained enriched in human iPSCs (Fig. 2B). Using thresholds of |log2 fold change| ≥ 1.0 and recalculated FDR < 0.05, we identified 9908 differentially expressed genes between human iPSCs and HLOs, including 5652 genes upregulated in HLOs and 4256 genes upregulated in human iPSCs. The complete list of differentially expressed genes is provided in Table S5. The MA plot also illustrated a marked transcriptional shift from a pluripotency-associated program toward one related to hepatic differentiation and metabolic function during HLO formation (Fig. 2C). GO analysis of genes upregulated in HLOs further supported this interpretation. In the BP category, terms related to xenobiotic stimulus response and lipid/steroid metabolic processes, steroid metabolism, and fatty acid metabolism were significantly enriched (Fig. 2D), indicating acquisition of hepatocyte-like metabolic and detoxification programs. In the CC category, enriched terms included endoplasmic reticulum lumen, apical plasma membrane, microvillus, and brush border membrane (Fig. 2E), consistent with the development of intracellular metabolic machinery and polarized epithelial organization. Together, these findings indicate that HLOs generated by the SC method predominantly possess hepatocyte-like properties while retaining partial biliary epithelial features. To improve readability, enlarged versions of the Fig. 2 panels are provided in Figs S2–S4 (see online supplementary material), including the heatmap, volcano and MA plots, and expanded GO enrichment results showing the top 20 terms in each category.

### Immunofluorescence analysis demonstrates hepatocyte-like and biliary epithelial-like components in HLOs generated by the SC method

**Figure 3:**
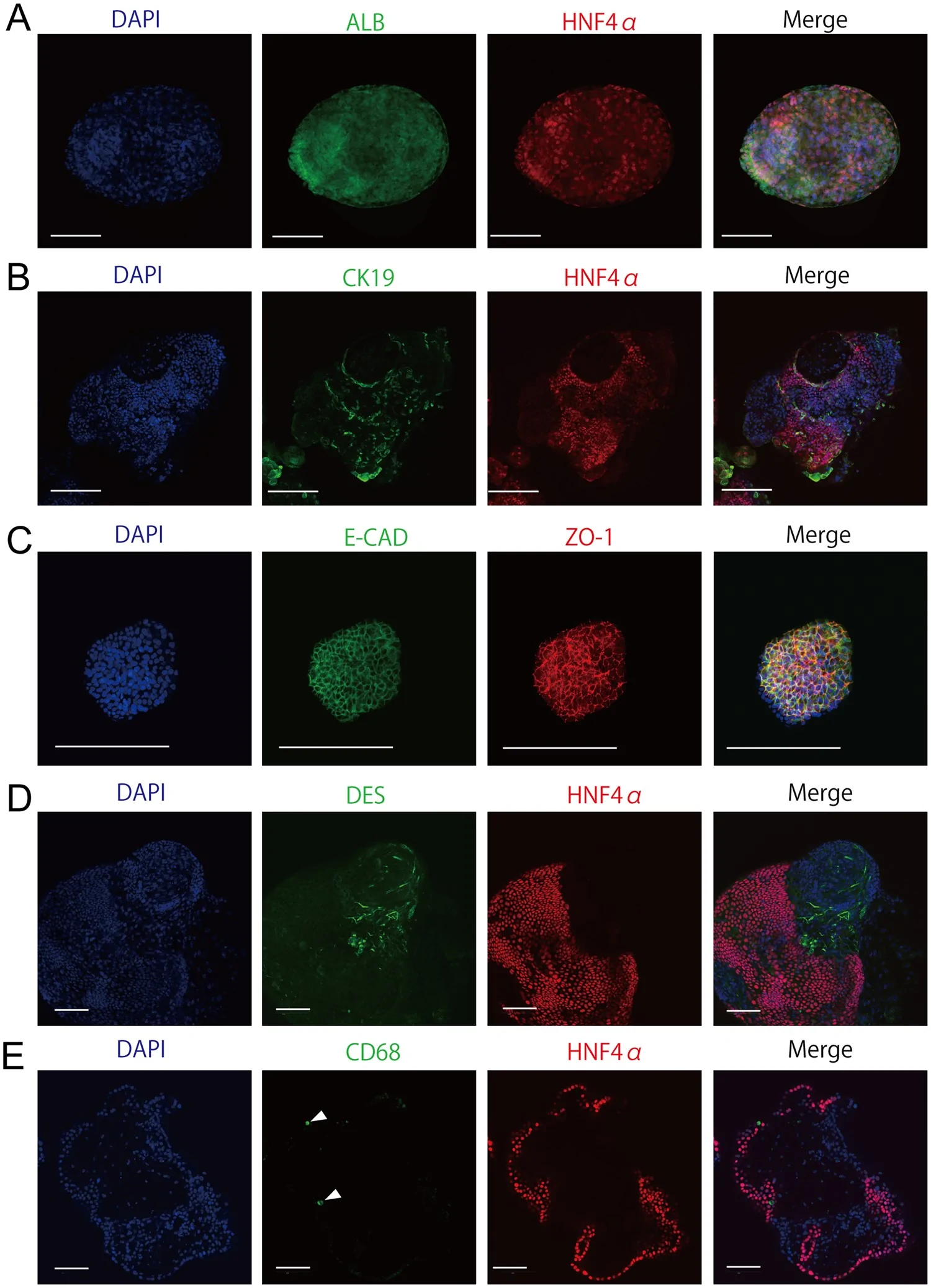
Immunofluorescence analysis of cellular composition in HLOs. (**A**–**E**) Representative immunofluorescence images of HLOs stained for the indicated markers. In all panels, nuclei were stained with DAPI (4′,6-diamidino-2-phenylindole); the leftmost images show DAPI staining alone, and the rightmost images show merged signals. (A) ALB (albumin) and HNF4α (hepatocyte nuclear factor 4 alpha). (B) CK19 (cytokeratin 19) and HNF4α. (C) E-CAD (E-cadherin) and ZO-1 (zonula occludens-1). (D) DES (desmin) and HNF4α. (E) CD68 (cluster of differentiation 68) and HNF4α. Arrowheads indicate representative CD68-positive cells. Scale bar, 100 µm.

To further define the cellular composition of HLOs generated by the SC method, we performed immunofluorescence staining. Strong signals for ALB and HNF4α were broadly detected throughout the organoids, indicating the presence of a prominent hepatocyte-like cell population (Fig. 3A). Because HNF4α is a key transcription factor in the hepatic lineage, its broad distribution together with ALB positivity supports the presence of liver parenchymal-like cells within the organoids. In addition, CK19-positive structures were observed adjacent to HNF4α-positive regions, suggesting the presence of biliary epithelial-like components (Fig. 3B). Furthermore, E-cadherin and ZO-1 staining was observed along epithelial cell boundaries in biliary epithelial-like structures. E-cadherin was interpreted as a marker of adherens junctions and cell–cell adhesion, whereas ZO-1 was interpreted as a tight junction-associated marker. These findings support the presence of epithelial junctional organization in biliary epithelial-like components within the organoids (Fig. 3C). These findings indicate that the organoids possess a tissue-like architecture containing not only hepatocyte-like components but also biliary epithelial-like elements. By contrast, DES-positive cells and CD68-positive cells were detected only in small numbers, suggesting that stromal-like or macrophage-like populations represent minor components in HLOs generated by the SC method (Fig. 3D and E). Taken together, these results indicate that HLOs generated by the SC method are composed predominantly of hepatic parenchymal-like and biliary epithelial-like structures with minor stromal-like and macrophage-like populations.

## Discussion

In this study, we established a simple suspension culture method for generating human iPSC-derived liver organoids without exogenous ECM scaffolds or specialized equipment. Organoids generated by this method exhibited sustained albumin secretion, relatively stable maintenance of CYP3A4 activity during extended culture (Fig. 1), and hepatic transcriptomic features characterized by upregulation of liver-associated transcription factors, functional genes, and drug metabolism-related genes (Fig. 2). Immunofluorescence analyses further demonstrated hepatocyte-like populations and biliary epithelial-like structures showing epithelial junctional organization, with only minor stromal-like and macrophage-like populations (Fig. 3). Taken together, these findings indicate that the present method enables the generation of organoids possessing key structural and functional features of human liver tissue while maintaining operational simplicity.

It should be noted that the present data do not indicate a simple superiority of either SC in terms of hepatic differentiation. Albumin secretion and CYP3A4 activity were clearly detected under the SC condition, supporting the acquisition of hepatocyte-like functions (Fig. 1). At the same time, the EE condition showed higher albumin secretion at several time points and a higher mean peak of CYP3A4 activity around day 30, whereas CYP3A4 activity subsequently declined. In contrast, the SC condition showed lower peak CYP3A4 activity but relatively stable activity during extended culture. Thus, although the possibility that SC-derived HLOs reach a lower maximal level of functional maturation cannot be excluded, these findings may also reflect differences in the temporal dynamics of functional maturation and maintenance between the SC and EE conditions.

The different CYP3A4 activity patterns between the EE and SC conditions may be explained, at least in part, by differences in ECM-mediated maturation cues and long-term culture microenvironments. Under the EE condition, Geltrex may promote hepatocyte-like maturation, thereby contributing to the higher peak activity around day 30. However, during prolonged gel-embedded culture, diffusion of oxygen, nutrients, assay substrates, and metabolites may become less uniform, potentially contributing to the subsequent decline in CYP3A4 activity. In contrast, the SC condition may provide weaker ECM-mediated maturation cues but allow more direct exposure of organoids to the culture medium, which may help maintain relatively stable CYP3A4 activity during extended culture. This possibility should be examined in future studies.

**Figure 4:**
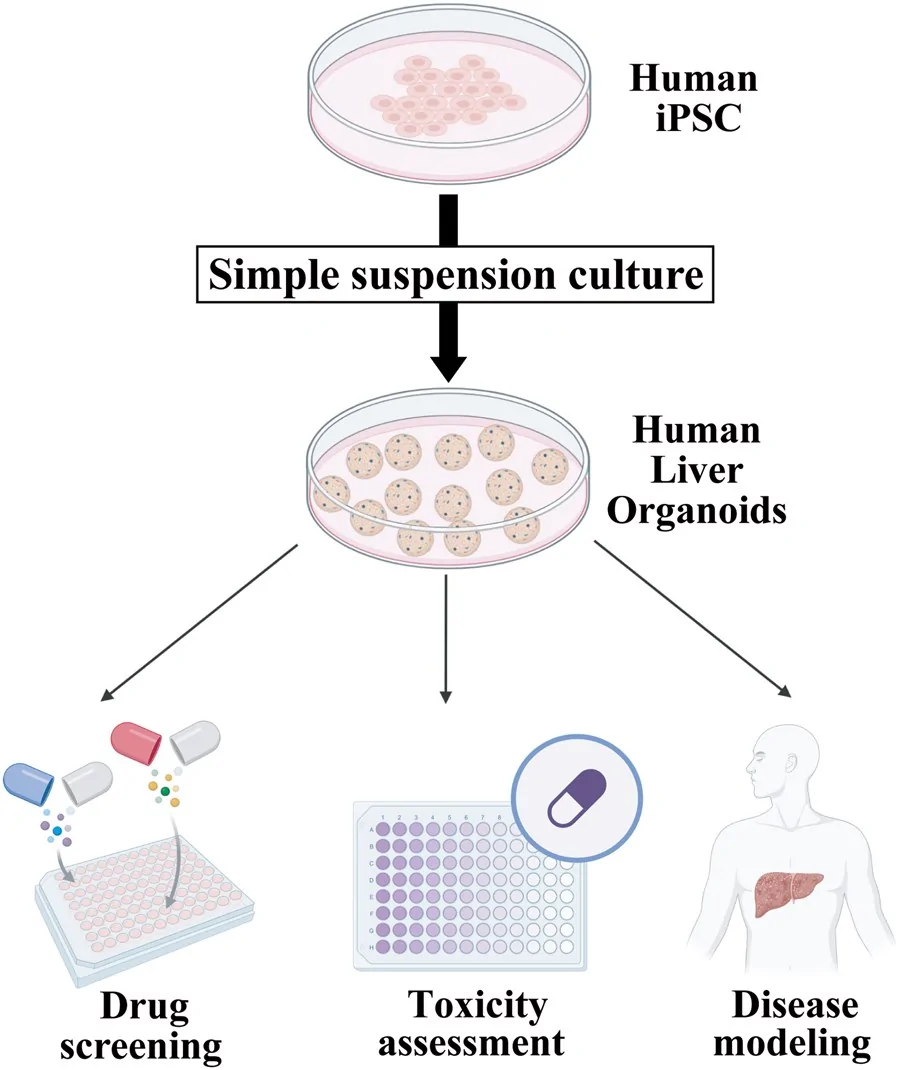
Schematic overview of the simple suspension culture method and potential applications of HLOs. Schematic diagram showing the generation of HLOs from human iPSCs through the simple suspension culture method and the potential downstream applications of the resulting HLOs, including drug screening, toxicity assessment, and disease modeling.

During preparation of this manuscript, further advances in HLO methodologies were reported, including a recent study describing vascularization- and maturation-promoting conditions in PSC-derived liver organoids [44]. In addition, alternative approaches that avoid conventional ECM embedding have been reported, including culture with low concentrations of ECM, self-organizing iPSC-derived liver organoids, and scalable suspension-based culture systems [32, 37, 38]. These studies underscore the growing interest in liver organoid methods that do not rely on standard ECM embedding. However, even when ECM embedding is avoided, many such approaches still require specialized culture formats, dedicated equipment, or technically demanding handling procedures to support organoid formation and maturation. By contrast, the method established in the present study enables organoid generation without exogenous ECM scaffolds, mechanical agitation, or specialized equipment. To clarify the practical positioning of the present SC method, we summarized representative human PSC-derived three-dimensional liver models in terms of model and culture format, requirement for exogenous ECM scaffolds, requirement for specialized culture equipment, and potential applications (Table 1). This practical simplicity of the present SC method, particularly its lack of requirement for exogenous ECM scaffolds or specialized culture equipment, is likely to be advantageous for broader implementation, especially in settings where reproducibility, scalability, and ease of adoption are important. Although direct generation of HLOs in 96- or 384-well plates remains to be optimized, organoids generated in bulk under identical SC conditions could be dispensed into multi-well plates for downstream assays. This feature may help reduce variability during organoid generation and facilitate future applications in drug screening, toxicity assessment, and disease modeling (Fig. 4).

**Table 1:** Methodological comparison of the present suspension culture method with representative human PSC-derived three-dimensional liver models.

Model and culture format	Exogenous ECM scaffold	Specialized equipment for culture	Potential applications	Reference(s)
**Present SC method: static suspension culture in conventional dishes**	Not required	Not required	Toxicity assessment, drug screening, disease modeling	This study
**Microfluidic liver-chip/MPS models with flow or perfusion**	Required	Required	DILI screening, long-term drug exposure studies	[15]
**Matrigel-based hPSC-derived hepatic organoids in droplet/suspension formats**	Required; reduced in suspension format	Not required	Drug safety assessment, CYP-mediated metabolism studies	[32]
**Multicellular hPSC-derived HLOs from foregut spheroids/progenitors**	Required	Not required	Steatohepatitis modeling, DILI/cholestasis screening	[21, 24]
**hPSC-derived hepato-biliary-pancreatic multi-organ organoids**	Required	Transwell ALI culture required	Hepato-biliary-pancreatic organogenesis modeling	[33]
**iPSC-derived hepatocyte-stellate cell co-culture organoids**	Not required	EZSPHERE SP microplate required	Hepatocyte-stellate cell interaction modeling	[29]
**Placenta factor-enhanced hiPSC-liver organoids**	Required	Controlled oxygen conditions required	Organoid growth, maturation, regenerative medicine	[30]
**Self-assembled Matrigel-free iPSC-derived liver organoids in RWVs**	Not required	RWV required	Drug metabolism, disease modeling, regenerative medicine	[37]
**Scalable vascularized hPSC-derived liver organoids in dynamic suspension culture**	Not required	Orbital shaker required	Scalable production, toxicity assessment, cell therapy	[39]
**Matrigel-free trilineage hiPSC-derived liver organoids with ALI culture**	Not required	ALI culture required	Liver organogenesis, vascularization, maturation modeling	[44]

Abbreviations: ALI, air–liquid interface; DILI, drug-induced liver injury; ECM, extracellular matrix; HLO, human liver organoid; hPSC, human pluripotent stem cell; hiPSC, human-induced pluripotent stem cell; MPS, microphysiological system; RWV, rotating wall vessel.

An important future direction for this method is its application to metabolism-dependent toxicity assessment. In addition to the detection of CYP3A4 activity, RNA-seq analysis showed increased expression of multiple drug metabolism-related genes in SC-derived HLOs compared with undifferentiated human iPSCs (Fig. 2). These included phase I CYP genes, such as CYP3A4, CYP3A5, CYP2C9, CYP2C19, CYP1A1, and CYP1B1, as well as phase II metabolism-related genes, including UGT1A1, UGT1A6, SULT1A1, and SULT1A2. These transcriptional features suggest that SC-derived HLOs acquire a hepatocyte-like metabolic gene expression program involving multiple drug metabolism-related pathways. This may be relevant for screening and toxicity assessment of compounds that are metabolized through diverse hepatic pathways. However, because CYP activity was directly measured only for CYP3A4 in the present study, future studies should evaluate the activities of additional CYP enzymes and other metabolic pathways to more fully define the metabolic capacity of SC-derived HLOs. Many toxic compounds require hepatic metabolic activation or are strongly influenced by hepatic detoxification pathways, making metabolically competent liver models essential for mechanistic evaluation. This is also relevant to genotoxicity research [45–49]. Although some DNA-damaging agents, including ionizing radiation [50–52], act independently of hepatic metabolism, many genotoxic compounds require metabolic activation or are substantially influenced by detoxification pathways [53]. In this context, recent work using HepaSH cells, a metabolically competent human hepatocyte-derived cell line, has shown that a two-dimensional system can detect DNA damage responses to metabolically activated genotoxicants such as benzo(a)pyrene, aristolochic acid, and PhIP under human-like metabolic conditions, further supporting the value of metabolically competent human liver models for genotoxicity assessment [54]. At the same time, three-dimensional models are expected to offer advantages for endpoints that depend on prolonged exposure, epithelial organization, multicellular architecture, or a more liver-like microenvironment. These considerations suggest that, rather than assuming that one model is uniformly superior in all settings, it will be important to compare different human liver models side by side, including two-dimensional hepatocyte systems and three-dimensional HLO-based systems, to determine which platforms are most suitable for particular toxicological endpoints. In this framework, HLOs generated by the present method may be particularly useful for evaluating toxicological responses that require prolonged exposure, epithelial organization, or multicellular liver-like architecture, whereas simpler two-dimensional models may remain advantageous for throughput and assay simplicity. Thus, systematic comparison of human liver models across different toxicological endpoints will be an important next step.

Several limitations of this study should be noted. First, the suspension culture method was evaluated using a single human iPSC line, ChiPSC18. Therefore, the applicability of this method to other human iPSC lines remains to be determined. Because differentiation propensity and organoid-forming capacity can vary among iPSC lines, future validation using multiple iPSC lines will be important to assess the robustness and general applicability of this method. Second, although the present study demonstrated albumin secretion, CYP3A4 activity, hepatic gene expression, and hepatocyte-like and biliary epithelial-like structures in SC-derived HLOs, the transcriptomic analysis was performed using SC-derived HLOs and undifferentiated human iPSCs, but not EE-derived HLOs. Therefore, the present data do not allow direct comparison of transcriptional maturation between SC- and EE-derived HLOs. In addition, the immunofluorescence analysis was not designed to quantitatively compare marker expression or tissue organization between the two SCs. Further direct comparisons with ECM-embedding methods and other established liver models, including transcriptional analysis of key hepatic genes and quantitative comparison of immunofluorescence markers, will be necessary to more precisely define the maturation status and suitable application range of SC-derived HLOs.

## Supplementary Material

bpag036_Supplementary_Data

## Data Availability

The raw sequencing data and processed RNA-seq data generated in this study have been deposited in the Gene Expression Omnibus under accession number GSE333276. The processed bulk RNA-seq data supporting the findings of this study, together with the downstream R scripts used for the transcriptomic analyses, are available in Zenodo: 10.5281/zenodo.19480024 [43].
