# Supplementary material for: A simple suspension culture method for generating human iPSC-derived liver organoids
Source: Biol Methods Protoc. 2026 Jun 25;11(1):bpag036. doi: 10.1093/biomethods/bpag036 (PMC13354523; doi:10.1093/biomethods/bpag036)
Supplement: bpag036_Supplementary_Data [file bpag036_supplementary_data.zip › Supplementary_Table3.pdf]

Supplementary Table 3. Raw data of albumin secretion

Day	Culture condition	Replicate	Albumin secretion (ng/mL/day)
7	SC	1	2.9025
7	SC	2	out of range
7	SC	3	out of range
7	EE	1	1.4895
7	EE	2	out of range
7	EE	3	1.5285
9	SC	1	2.9250
9	SC	2	0.6645
9	SC	3	0.1005
9	EE	1	0.9600
9	EE	2	0.2445
9	EE	3	out of range
11	SC	1	0.6750
11	SC	2	2.0970
11	SC	3	0.8550
11	EE	1	out of range
11	EE	2	0.0825
11	EE	3	4.0110
13	SC	1	1.9890
13	SC	2	1.5450
13	SC	3	0.2265
13	EE	1	1.6020
13	EE	2	4.9485
13	EE	3	out of range
16	SC	1	0.8800
16	SC	2	2.3250
16	SC	3	2.1490
16	EE	1	49.6440
16	EE	2	48.2890
16	EE	3	52.9710
19	SC	1	7.4960
19	SC	2	7.1790
19	SC	3	4.2990
19	EE	1	351.9490
19	EE	2	342.3630
19	EE	3	325.7900
22	SC	1	19.9190
22	SC	2	27.5030
22	SC	3	26.9340
22	EE	1	287.7400
22	EE	2	294.6840
22	EE	3	237.7690
25	SC	1	62.7830
25	SC	2	66.0280
25	SC	3	48.8430
25	EE	1	863.5370
25	EE	2	477.5910
25	EE	3	365.0330
28	SC	1	260.2370
28	SC	2	214.9470
28	SC	3	221.5060
28	EE	1	343.7030
28	EE	2	320.9850
28	EE	3	239.8610
31	SC	1	69.5730
31	SC	2	61.0760
31	SC	3	71.0740
31	EE	1	131.6580
31	EE	2	265.1850
31	EE	3	237.0790
34	SC	1	54.9790
34	SC	2	59.9000
34	SC	3	70.2620
34	EE	1	347.7810
34	EE	2	279.2360
34	EE	3	163.1660
37	SC	1	48.7640
37	SC	2	52.4590
37	SC	3	59.0340
37	EE	1	159.5470
37	EE	2	92.7980
37	EE	3	71.7790
40	SC	1	25.6160
40	SC	2	32.6590
40	SC	3	39.3430
40	EE	1	154.6910
40	EE	2	79.1670
40	EE	3	157.7860
43	SC	1	22.3310
43	SC	2	38.0360
43	SC	3	22.6590
43	EE	1	118.4470
43	EE	2	100.6620
43	EE	3	104.1720
46	SC	1	54.5360
46	SC	2	21.5900
46	SC	3	22.8470
46	EE	1	42.2740
46	EE	2	52.9710
46	EE	3	36.2610
50	SC	1	18.8760
50	SC	2	9.9368
50	SC	3	8.7893
50	EE	1	20.6273
50	EE	2	20.8620
50	EE	3	14.3775
