# Supplementary material for: A simple suspension culture method for generating human iPSC-derived liver organoids
Source: Biol Methods Protoc. 2026 Jun 25;11(1):bpag036. doi: 10.1093/biomethods/bpag036 (PMC13354523; doi:10.1093/biomethods/bpag036)
Supplement: bpag036_Supplementary_Data [file bpag036_supplementary_data.zip › Supplementary_Table4.pdf]

Supplementary Table 4. Raw data of CYP3A4 activity

day	SC1	SC2	SC3	EE1	EE2	EE3
23	631.486	569.401	798.437	648.817	581.253	735.742
30	1275.036	1328.966	1468.232	4155.900	4355.310	2401.893
40	1210.826	1570.951	1239.739	4046.032	2568.962	3172.500
50	1316.144	1716.037	1964.375	2139.141	1867.491	903.863
