# Supplementary material for: A simple suspension culture method for generating human iPSC-derived liver organoids
Source: Biol Methods Protoc. 2026 Jun 25;11(1):bpag036. doi: 10.1093/biomethods/bpag036 (PMC13354523; doi:10.1093/biomethods/bpag036)
Supplement: bpag036_Supplementary_Data [file bpag036_supplementary_data.zip › Supplementary_Table6.pdf]

Supplementary Table 6. Statistical analysis of albumin secretion and CYP3A4 activity

Two-way ANOVA

Figure panel	Measurement	Effect	Df	F value	P value	Significance
Fig. 1D	Albumin secretion	condition	1.000	110.936	4.38e-15	**
Fig. 1D	Albumin secretion	day	15.000	16.497	1.19e-15	**
Fig. 1D	Albumin secretion	condition × day	15.000	9.507	1.05e-10	**
Fig. 1E	CYP3A4 activity	condition	1.000	22.548	0.000218	**
Fig. 1E	CYP3A4 activity	day	3.000	14.270	8.73e-05	**
Fig. 1E	CYP3A4 activity	condition × day	3.000	7.918	0.001841	**

Bonferroni-adjusted post hoc comparisons between SC and EE at each time point

Figure panel	Measurement	Day	Contrast	Estimate	SE	df	t ratio	Bonferroni-adjusted P value	Significance
Fig. 1D	Albumin secretion	7	SC - EE	1.393	68.885	58	0.02	1.0000	ns
Fig. 1D	Albumin secretion	9	SC - EE	0.628	51.344	58	0.012	1.0000	ns
Fig. 1D	Albumin secretion	11	SC - EE	-0.838	51.344	58	-0.016	1.0000	ns
Fig. 1D	Albumin secretion	13	SC - EE	-2.022	51.344	58	-0.039	1.0000	ns
Fig. 1D	Albumin secretion	16	SC - EE	-48.517	45.923	58	-1.056	1.0000	ns
Fig. 1D	Albumin secretion	19	SC - EE	-333.709	45.923	58	-7.267	<0.0001	**
Fig. 1D	Albumin secretion	22	SC - EE	-248.612	45.923	58	-5.414	<0.0001	**
Fig. 1D	Albumin secretion	25	SC - EE	-509.502	45.923	58	-11.095	<0.0001	**
Fig. 1D	Albumin secretion	28	SC - EE	-69.286	45.923	58	-1.509	1.0000	ns
Fig. 1D	Albumin secretion	31	SC - EE	-144.066	45.923	58	-3.137	0.0429	*
Fig. 1D	Albumin secretion	34	SC - EE	-201.681	45.923	58	-4.392	0.0008	**
Fig. 1D	Albumin secretion	37	SC - EE	-54.622	45.923	58	-1.189	1.0000	ns
Fig. 1D	Albumin secretion	40	SC - EE	-98.009	45.923	58	-2.134	0.5931	ns
Fig. 1D	Albumin secretion	43	SC - EE	-80.085	45.923	58	-1.744	1.0000	ns
Fig. 1D	Albumin secretion	46	SC - EE	-10.844	45.923	58	-0.236	1.0000	ns
Fig. 1D	Albumin secretion	50	SC - EE	-6.088	45.923	58	-0.133	1.0000	ns
Fig. 1E	CYP3A4 activity	23	SC - EE	11.200	438.294	16	0.025	1.0000	ns
Fig. 1E	CYP3A4 activity	30	SC - EE	-2280.300	438.294	16	-5.203	0.0003	**
Fig. 1E	CYP3A4 activity	40	SC - EE	-1922.000	438.294	16	-4.385	0.0018	**
Fig. 1E	CYP3A4 activity	50	SC - EE	28.700	438.294	16	0.065	1.0000	ns
