# Supplementary material for: A simple suspension culture method for generating human iPSC-derived liver organoids
Source: Biol Methods Protoc. 2026 Jun 25;11(1):bpag036. doi: 10.1093/biomethods/bpag036 (PMC13354523; doi:10.1093/biomethods/bpag036)
Supplement: bpag036_Supplementary_Data [file bpag036_supplementary_data.zip › Supplementary Table1.pdf]

Supplementary Table 1. Culture media and supplements used in this study

Stage	Supplement / Additive	Manufacturer	Catalog number
Definitive endoderm induction	Recombinant Human/Mouse/Rat Activin A	R&D Systems	338-AC
Definitive endoderm induction	Recombinant Human Bone Morphogenetic Protein 4 (BMP-4)	Proteintech	HZ-1045
Definitive endoderm induction	Recombinant Human Fibroblast Growth Factor 4 (FGF-4), Animal-Free	PeproTech	AF-100-31
Definitive endoderm induction	CHIR 99021, GSK-3 Inhibitor	R&D Systems	4423
HLO induction (RA stage)	all-trans Retinoic Acid	Sigma-Aldrich	R2625
HLO maturation	Recombinant Human Hepatocyte Growth Factor (HGF)	PeproTech	100-39
HLO maturation	Recombinant Human Oncostatin M	PeproTech	300-10H
HLO maturation	Dexamethasone	Sigma-Aldrich	D4902
Matrix (alternative)	Geltrex™ Reduced-Growth Factor Basement-Membrane Matrix, LDEV-free, stem-cell qualified	Gibco	A1413302
Basal medium	RPMI 1640 Medium	Gibco	11875093
Basal medium	Advanced DMEM/F12	Gibco	12634010
Basal medium	PowerPrimary HEP Medium (PPM)	Cellartis	Y20020
Supplement	B-27 Supplement (50×)	Gibco	17504044
Supplement	N-2 Supplement (100×)	Gibco	17502048
Supplement	HEPES Buffer Solution (1 M)	Gibco	15630080
Supplement	L-Glutamine Solution (200 mM, 100×)	FUJIFILM Wako	073-05391
Supplement	KnockOut™ Serum Replacement	Gibco	10828028
