# Supplementary material for: A simple suspension culture method for generating human iPSC-derived liver organoids
Source: Biol Methods Protoc. 2026 Jun 25;11(1):bpag036. doi: 10.1093/biomethods/bpag036 (PMC13354523; doi:10.1093/biomethods/bpag036)
Supplement: bpag036_Supplementary_Data [file bpag036_supplementary_data.zip › Supplementary Table2.pdf]

Supplementary Table 2. List of antibodies used in this study

Antibody	Cat#	RRID	Supplier	Dilution
HNF-4-alpha Rabbit Monoclonal antibody	ab200142	Not identified	Abcam	1:1000
Cytokeratin 19 Mouse Monoclonal antibody	sc6278	AB_627851	Santa Cruz	1:500
ZO-1 Mouse Monoclonal antibody	610966	AB_398279	BD Biosciences	1:500
E-Cadherin Goat polyclonal antibody	AF648	AB_355504	R&D Systems	1:500
Desmin Mouse Monoclonal antibody	ab8470	AB_306577	Abcam	1:100
CD68 Goat Monoclonal antibody	ab289671	Not identified	Abcam	1:1000
Donkey Anti-Mouse IgG H&L (Alexa Fluor® 488)	ab150105	AB_2732856	Abcam	1:200
Donkey Anti-Goat IgG H&L (Alexa Fluor® 488)	A32814	AB_2762838	Invitrogen	1:200
Donkey Anti-Rabbit IgG H&L (Alexa Fluor® 555)	A32794	AB_2762834	Invitrogen	1:200
Donkey Anti-Mouse IgG H&L (Alexa Fluor® 555)	A32773	AB_2762848	Invitrogen	1:200
