# Supplementary figures and images for: A simple suspension culture method for generating human iPSC-derived liver organoids
Source: Biol Methods Protoc. 2026 Jun 25;11(1):bpag036. doi: 10.1093/biomethods/bpag036 (PMC13354523; doi:10.1093/biomethods/bpag036)

Figure S1

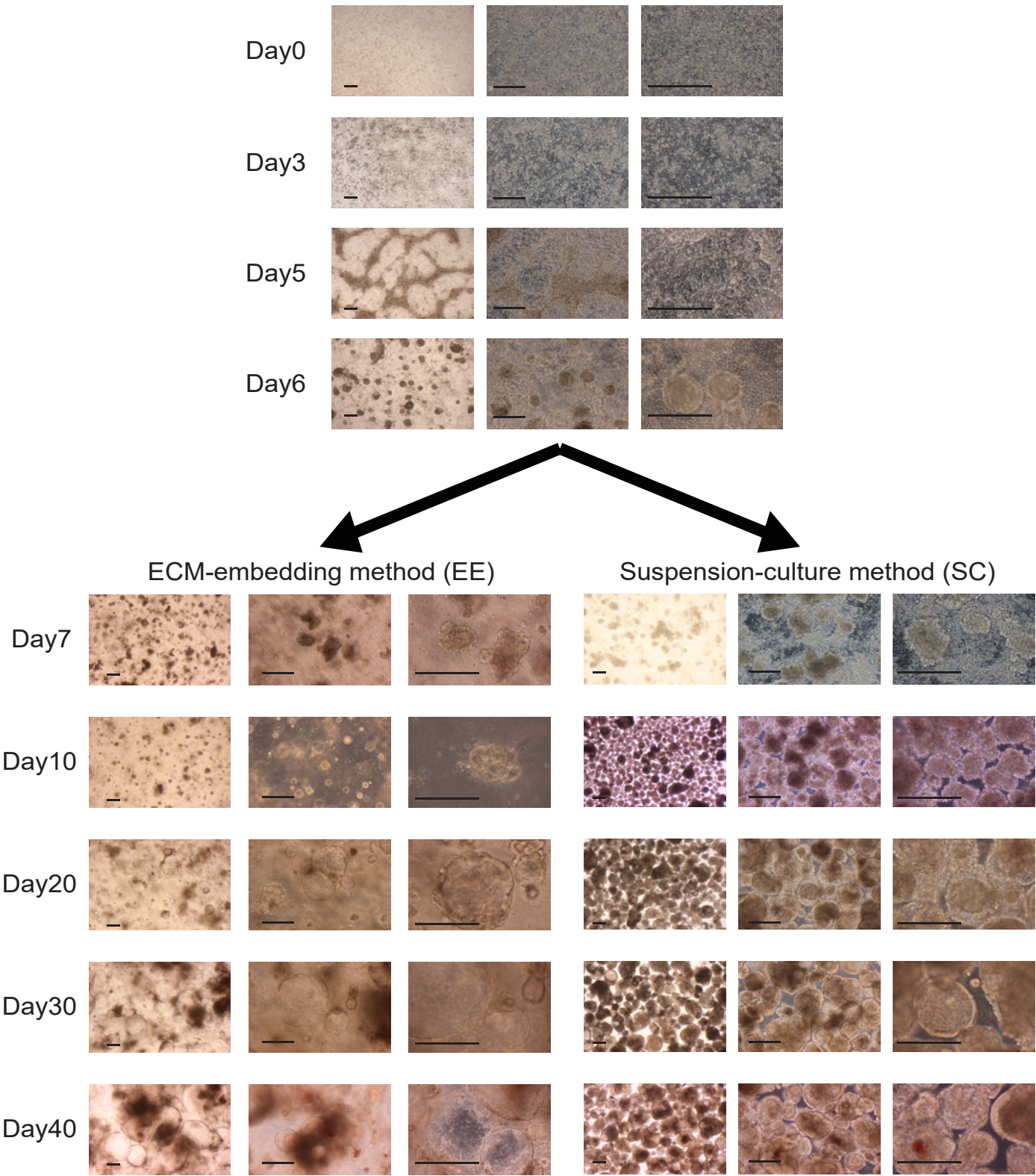

Figure S2

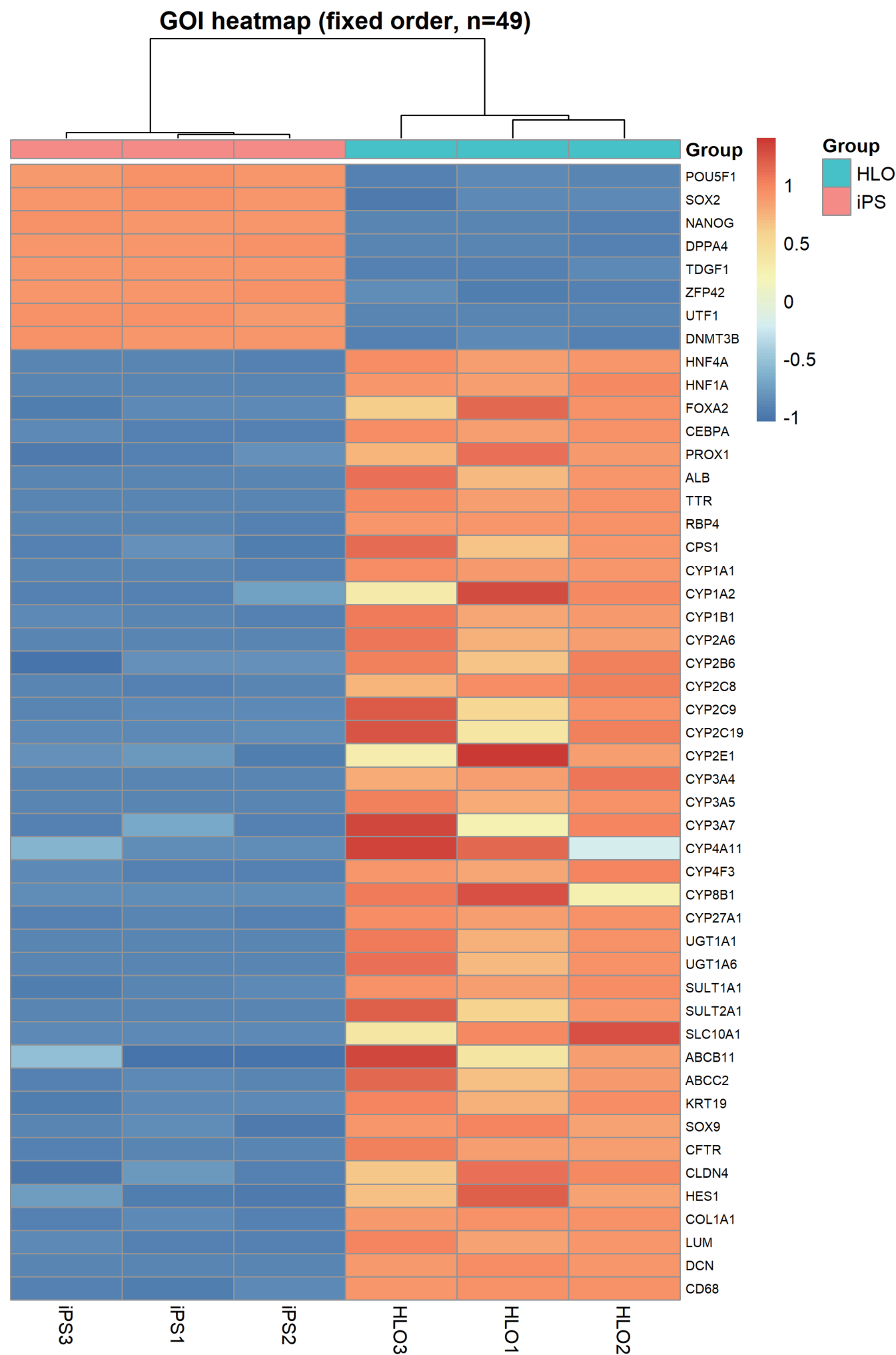

Figure S3

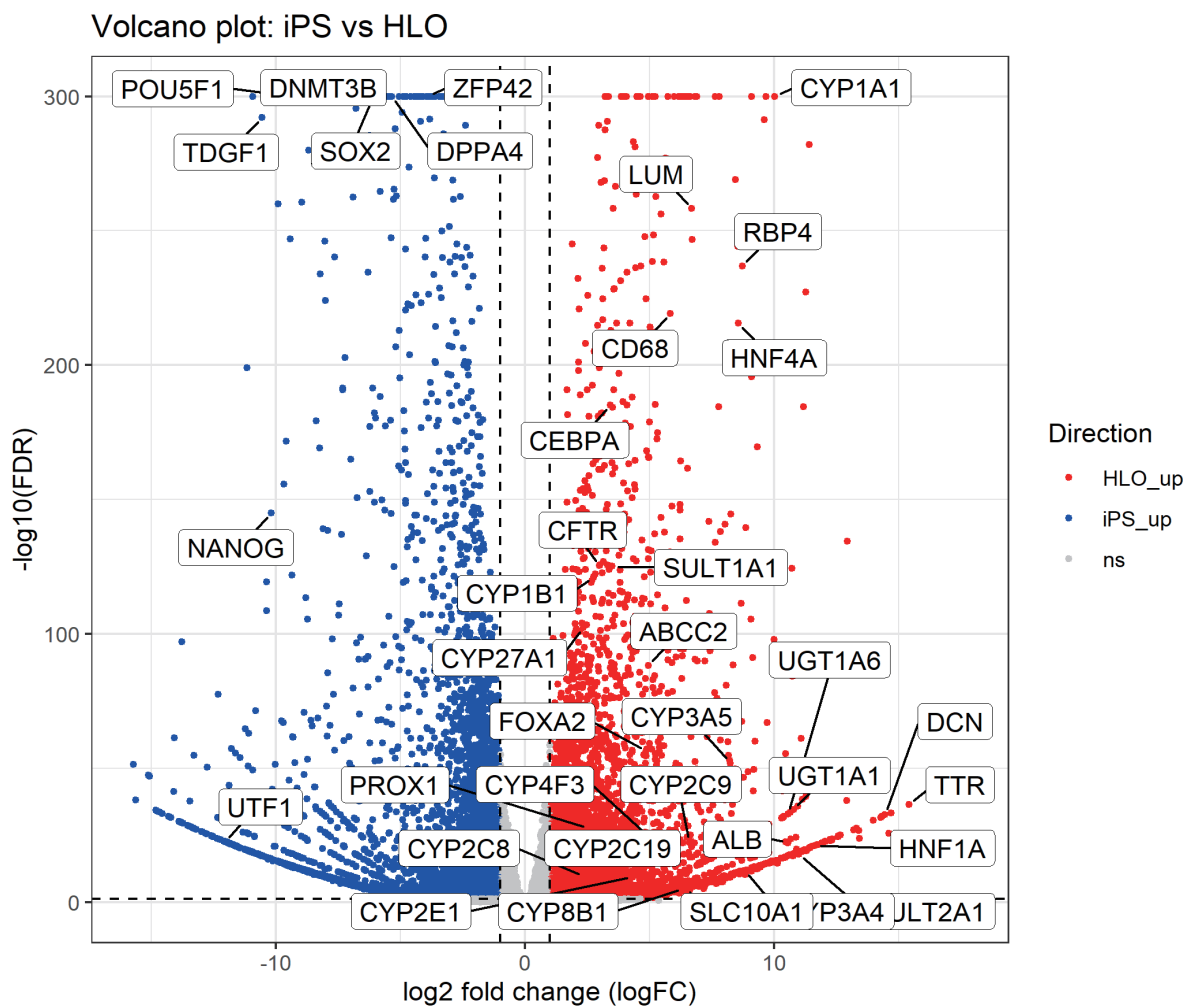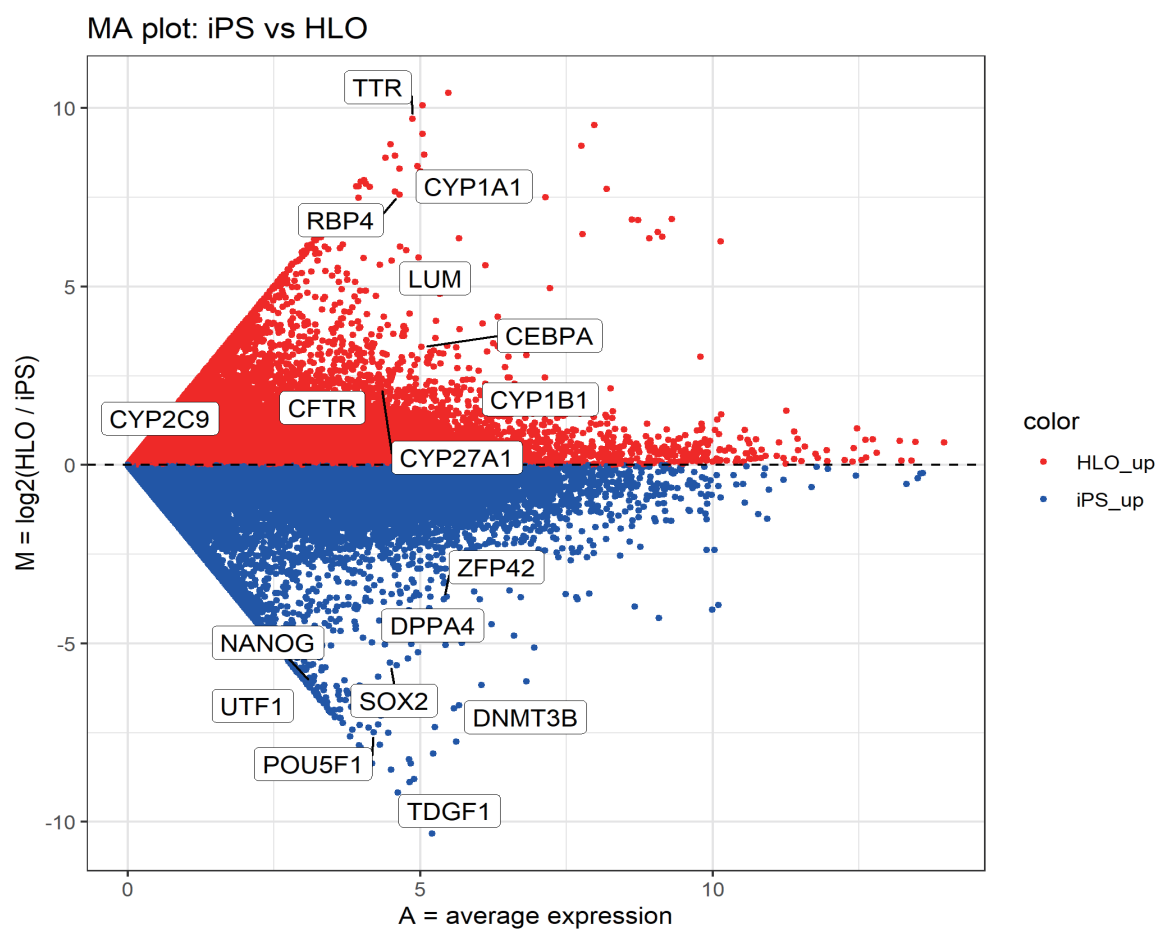

Figure S4

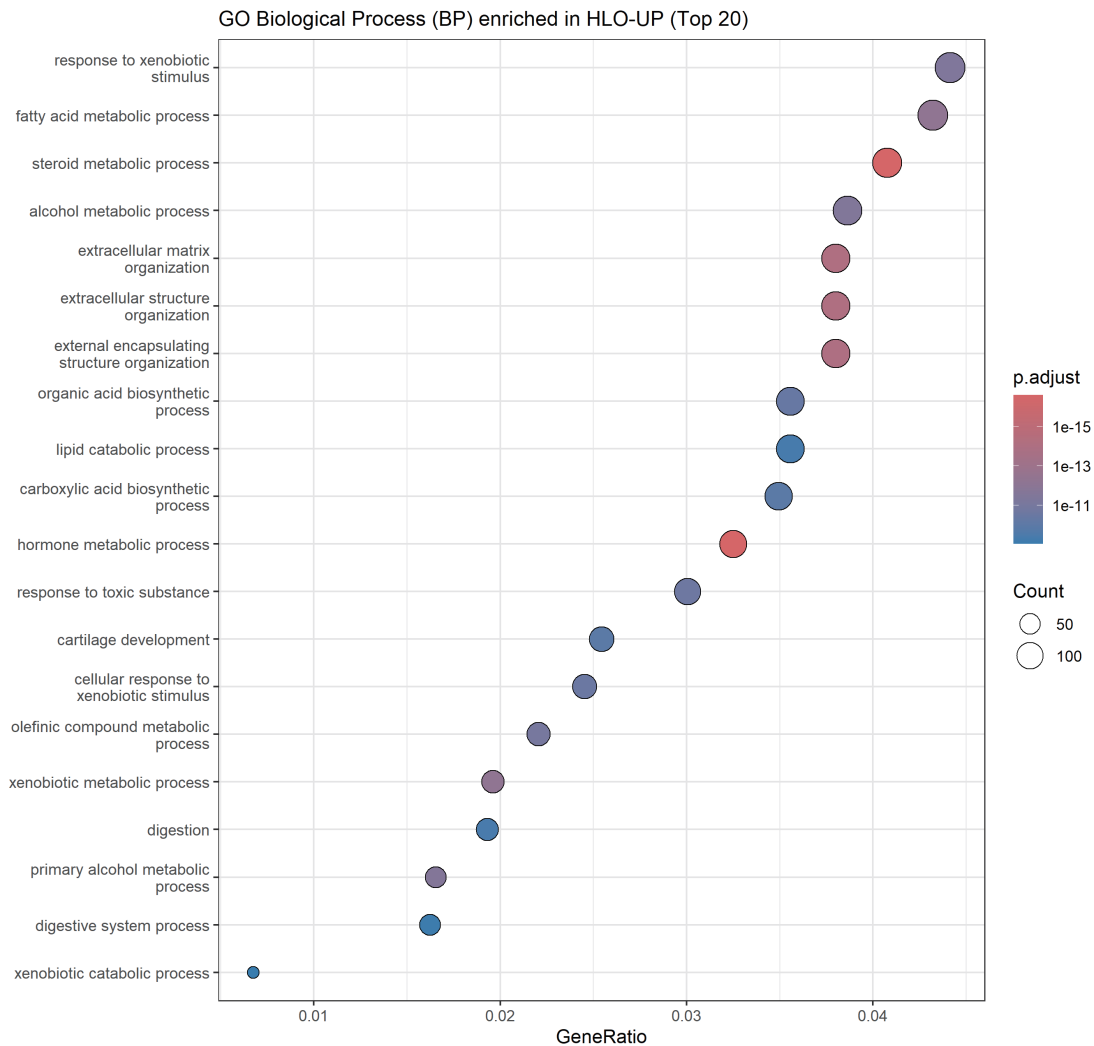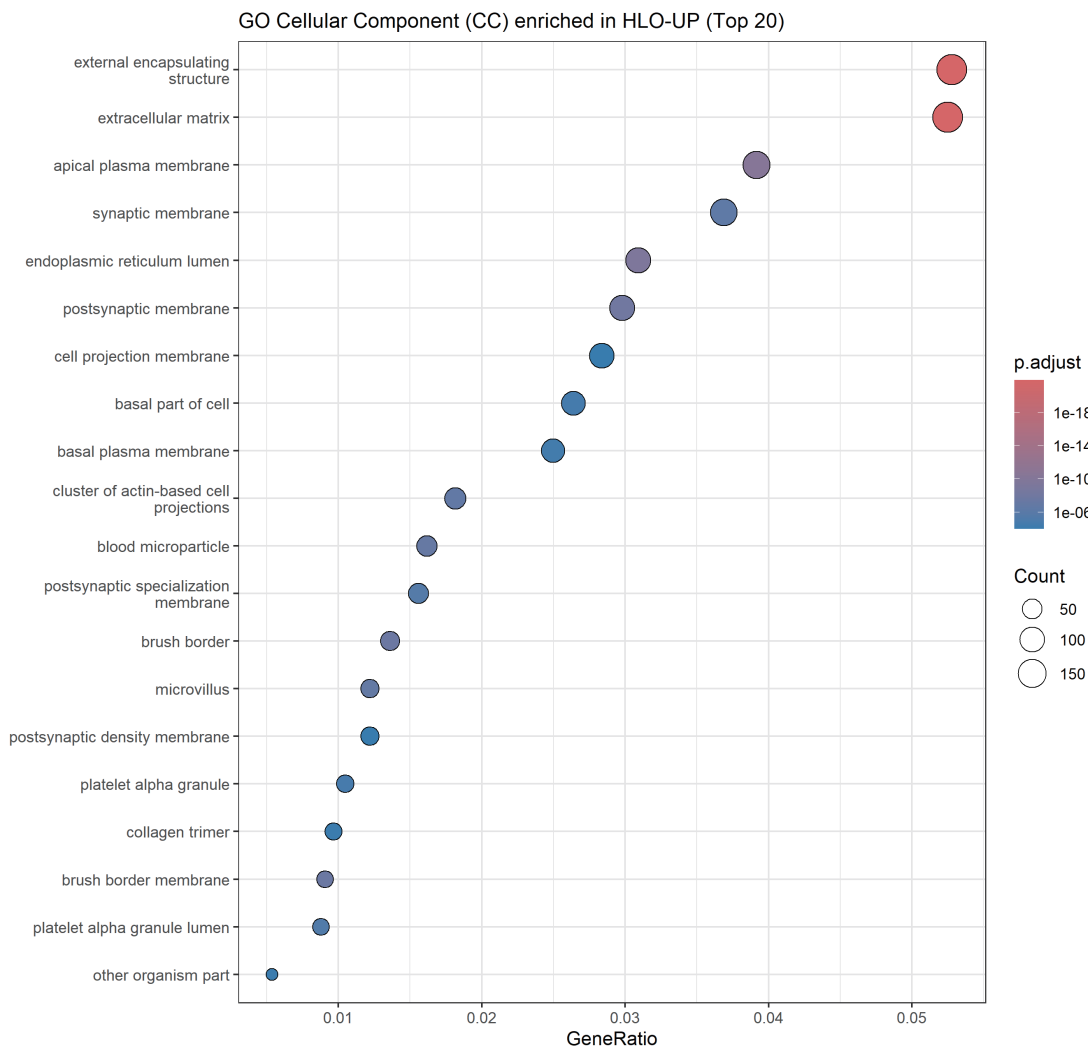

Supplement: bpag036_Supplementary_Data [file bpag036_supplementary_data.zip › Figures S_final_merged.pdf]
